# Transcriptome and Metabolome Analyses of Aroma Differences between Chardonnay and a Chardonnay Bud Sport

**DOI:** 10.3390/molecules29153671

**Published:** 2024-08-02

**Authors:** Xiaoqin Bao, Jin Dong, Min Niu, Zhilei Wang, Guoqian Xu

**Affiliations:** 1 College of Wine and Horticulture, Ningxia University, Yinchuan 750021, China; 12022131362@stu.nxu.edu.cn (X.B.); 12023131657@stu.nxu.cn (J.D.); nm970804@163.com (M.N.); 2 Engineering Research Center of Grape and Wine, Ministry of Education, Yinchuan 750021, China; 3 Ningxia Grape and Wine Engineering Technology Center, Yinchuan 750021, China

**Keywords:** ‘Chardonnay’ bud sport, SPME−GC−MS, muscat aroma, transcriptome

## Abstract

Chardonnay is one of the most popular white grape wine varieties in the world, but this wine lacks typical aroma, considered a sensory defect. Our research group identified a Chardonnay bud sport with typical muscat characteristics. The goal of this work was to discover the key candidate genes related to muscat characteristics in this Chardonnay bud sport to reveal the mechanism of muscat formation and guide molecular design breeding. To this end, HS−SPME−GC−MS and RNA−Seq were used to analyze volatile organic compounds and the differentially expressed genes in Chardonnay and its aromatic bud sport. Forty-nine volatiles were identified as potential biomarkers, which included mainly aldehydes and terpenes. Geraniol, linalool, and phenylacetaldehyde were identified as the main aroma components of the mutant. The GO, KEGG, GSEA, and correlation analysis revealed *HMGR*, *TPS1*, *TPS2*, *TPS5*, *novel.939*, and *CYP450* as key genes for terpene synthesis. *MAO1* and *MAO2* were significantly downregulated, but there was an increased content of phenylacetaldehyde. These key candidate genes provide a reference for the development of functional markers for muscat varieties and also provide insight into the formation mechanism of muscat aroma.

## 1. Introduction

Chardonnay is one of the most popular white grape wine varieties and is widely cultivated around the world [1]. However, the lack of a typical aroma of its berry results in a wine lacking in aroma. Bud sports are common variations that can occur in the natural growth of grapes, and these can be used to obtain improved varieties by modifying individual traits without changing the desirable qualities of the parent plant [2]. Bud sport selection is an important grape variety selection technique that has been used to cultivate varieties with desired traits, such as shorter ripening cycles, larger berries, and different berry colors [3]. However, there have been no reports of bud sports producing berries with altered aromas. Further, the underlying regulatory mechanisms of aroma remain poorly understood. Our research group found a bud sport of Chardonnay that produces fruit with a strong aroma but is not significantly different in other biological traits. Aroma is an important intrinsic quality of grape and wine and a key contributor to the overall sensory experience of wine. According to the aroma characteristics, we divided the grapes into muscat varieties like *V. vinifera* cv. Muscat blanc and cv. Muscat of Alexandria; non-muscat aromatic varieties, e.g., *V. vinifera* cv. Riesling; and neutral varieties such as *V. vinifera* cv. Chardonnay [4]. Chardonnay is considered a ‘neutral’ grape variety, so Chardonnay wine is typically fermented in oak barrels to obtain a pleasant aroma, though this greatly increases production costs. Thus, it is necessary to breed new varieties or strains of grapes with better aromas to promote the development of grape and wine industrialization in China.

Recent studies of grape aroma have focused on detecting differences in volatile compounds (VOCs) and the evolution of these VOCs during grape berry ripening [5,6]. There are many kinds of aroma substances in grapes, including terpenes, aldehydes, alcohols, ketones, C13-norisoprenoids, and esters [7], according to their biosynthetic origins as fatty acid derivatives, amino acid derivatives, terpenoids, and phenylpropanoids/benzenoids [8]. Terpenes, such as geraniol, linalool, nerol, α-terpineol, and citronellol, have been identified as the main contributors to the characteristic aroma of muscat varieties [5]. The main aroma components of muscat grapes and wine are produced by the mevalonate (MVA) and methyl pentose (MEP) pathways. The MVA pathway mainly synthesizes sesquiterpene and triterpene in the cytoplasm, and the MEP pathway mainly synthesizes monoterpene, diterpene, and tetraterpene in the plastid. Although there is spatial separation of plastids and cytoplasm in plants, the MVA pathway can also synthesize monoterpenes [9,10]. The odor of phenylacetaldehyde is often described as a rich rose fragrance, and its threshold is low, so this compound plays an important role in the formation of grape characteristic aroma [11]. Phenylacetaldehyde is produced by the deamination and decarboxylation of L-phenylalanine, and subsequent reduction leads to the formation of phenylethanol [12]. Decarboxylase, monoamine oxidase (MAO), and aryl alcohol dehydrogenase (AALDH) are the three key enzymes involved in converting phenylalanine to phenylacetaldehyde and phenylethanol [13]. Although some studies have investigated the types, sources, and influencing factors of grape aroma substances, there has been little in-depth research on the grape aroma formation mechanism.

The main purpose of this study was to explore the possible causes of muscat formation in grape berries by comparing Chardonnay with a muscat-scented bud sport, CH09. We compared the volatile organic compounds (VOCs) in CH and CH09 and identified the main odor-contributing volatile components. Next, we conducted global expression analysis using RNA-Seq to identify the key candidate genes controlling grape aroma and used qPCR to validate our findings. The results will be helpful for understanding the formation of aroma in the bud mutant fruit and the pathways of grape aroma formation and can guide the generation of improved resources for the breeding of new varieties.

## 2. Results

### 2.1. Differences in the Genetic Background and Physiological and Biochemical Indicators between Chardonnay and a Chardonnay Bud Sport

The genetic identification of CH09 as a bud sport of CH was carried out using the ISSR technique, and the genetic relationship between CH and CH09 was assessed by gel analysis (Figure S1A). NTSYS2.10e software was used for cluster analysis (Figure S1B). The results showed a similar genetic background for CH and CH09, with a similarity coefficient of 0.85. This further confirms that CH09 is a bud mutant of CH.

**Figure 1:**
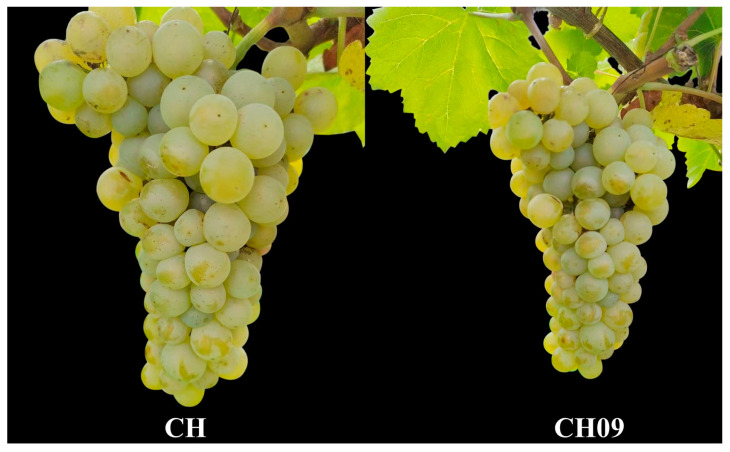
Fruit characterization of CH and CH09 at the mature stage.

The physiological properties of CH and CH09 were compared. There was no significant difference between CH and CH09 in the buds, leaves, and branches (Figure S2). The cluster of CH had secondary shoots, but CH09 had no secondary shoots (Figure 1).

As shown in Table S1, the cluster longitudinal diameter of CH and CH09 were 135.09 mm and 124.10 mm, respectively. The cluster longitudinal diameter of CH09 was significantly smaller than that of CH. The single berry weight of CH09 was significantly higher than that of CH, and the transverse diameters of the single berry were 10.51 mm and 10.96 mm, respectively, a significant difference. In general, the CH09 cluster was smaller than the CH cluster, but the CH09 single berry was larger than the CH single berry.

As shown in Table S2, the reducing sugar content of CH09 was significantly lower than that of CH, with values of 184.3 g/L and 192.17 g/L, respectively. The tannins of CH peel and seed were 20.01 mg/g and 34.12 mg/g, respectively, and those of CH09 were 22.38 mg/g and 34.94 mg/g, respectively. The amount of peel tannins was significantly higher for CH09 than for CH.

### 2.2. Volatile Metabolite Analysis of CH and CH09

#### 2.2.1. Overview of VOC Profiles in CH and CH09

**Figure 2:**
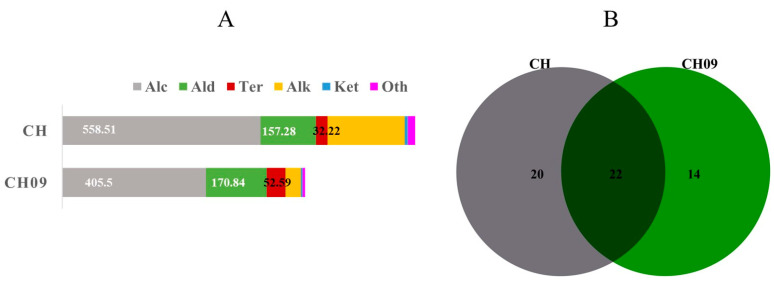
Volatile profiles of CH and CH09. The total concentration of each class (**A**) and the Venn diagram of VOCs (**B**).

The composition and relative content of aroma compounds in CH and CH09 were analyzed and identified by HS−SPME and GC−MS. As shown in Table S3, a total of 57 compounds were detected in CH09 and CH, including 18 alcohols, 14 alkanes, 12 aldehydes, 7 terpenes, 4 ketones, and 2 other compounds. Compared with CH, the total concentrations of volatile compounds in CH09 were much lower than those of CH, and the total contents of alcohols, alkanes, ketones, and other classes were also lower than those in CH, but the contents of terpenes (52.59 μg/L) and aldehydes (170.84 μg/L) were higher than those of CH (Figure 2A). A total of 42 VOCs were detected in CH, and 36 VOCs were detected in CH09. CH had 20 unique aroma compounds, accounting for 35.08% of the total number of compounds. Fourteen compounds were unique to CH09, accounting for 25% of the total compounds. Twenty-two VOCs were found in both samples, accounting for 38.60% (Figure 2B).

#### 2.2.2. Principal Component and Multivariate Analysis of Differential VOCs

**Figure 3:**
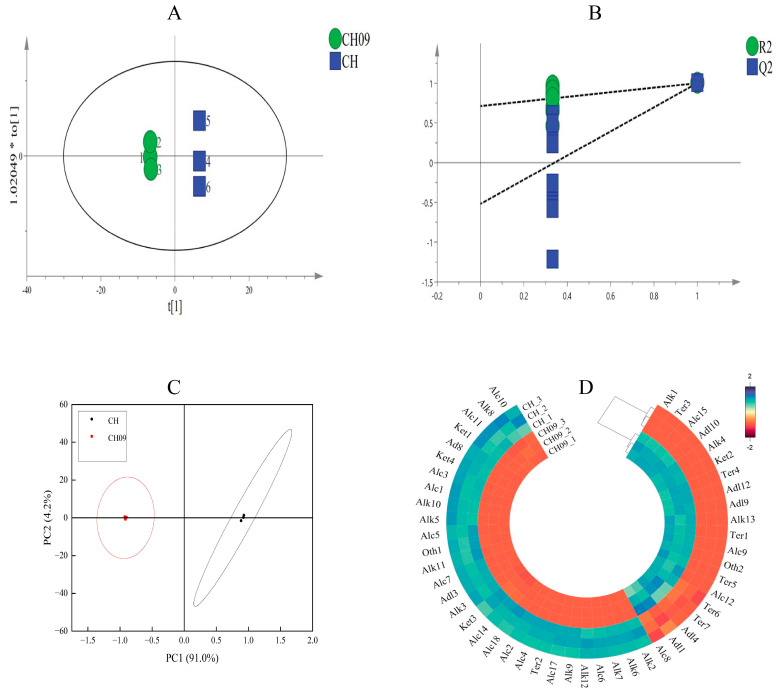
Principal component and multivariate analysis of differential VOCs. The OPLS−DA plot of CH09 (green) and CH (blue) (**A**), permutation test of OPLS−DA model (**B**), PCA plot of CH and CH09 (**C**), and cluster heat map of different aroma components in CH09 and CH grape fruits (**D**).

We explored the specific characteristics of VOC profiles by OPLS−DA [14]. As shown in Figure 3A, the 57 volatiles can effectively distinguish CH and CH09. After 200 permutation tests, the intersection of the Q^2^ regression line and the longitudinal axis was less than 0 (Figure 3B), indicating that the model is valid, and the results can be used to distinguish CH and CH09. The percentage of variation explained by PC1 and PC2 in the principal components analysis (PCA) was 95.20% (PC1 + PC2), so these two PCs represented the main characteristics of the sample (Figure 3C). To determine the contribution of each VOC in distinguishing CH and CH09, VIP (variable importance of projection) values were calculated for the 57 VOCs, and values ≥ 1 are given in Table S3. Forty-nine VOCs were identified with *p* < 0.05 and VIP ≥ 1, as illustrated in Figure 3D. In addition, phenylacetaldehyde was detected in CH09 but not in CH.

VOCs can be sensed and tasted directly and contribute to the aroma of grape berries at concentrations above the odor threshold (OAV ≥ 1) [15]. In this study, the OAVs were calculated for terpenes and phenylacetaldehyde. In general, the contents of terpenes and aldehydes in CH09 were higher than those in CH, and geraniol, linalool, and phenylacetaldehyde were the most abundant compounds. In CH09, the OAVs of geraniol, linalool, and phenylacetaldehyde were 3.06, 36.16, and 5.45, respectively (Table S4). Overall, the different kinds of terpenoids and the different relative abundances of terpenoids explain the difference in aroma between CH and CH09.

### 2.3. Transcription Sequencing Analysis

#### 2.3.1. Identification of Differentially Expressed Genes (DEGs)

To study the molecular mechanisms underlying the differences in aromatic volatile metabolites, we next performed transcriptome analysis of mature grape berries from Chardonnay and its mutant. A total of 37.25 G clean data were obtained, with at least 6.04 G for each sample, Q20 ≥ 97.32%, Q30 ≥ 93%, GC content of 46.64%~45.16%, and an overall sequencing error rate less than 0.03%, indicating good quality sequencing data. The mapping ratio was obtained by sequence alignment, with 2.96%~3.27% multiply mapped reads and 85.36%~87.01% uniquely mapped reads. The six samples were consistent with the reference genome, and the repeatability was high, indicating the good accuracy of the experiment (Table S5).

**Figure 4:**
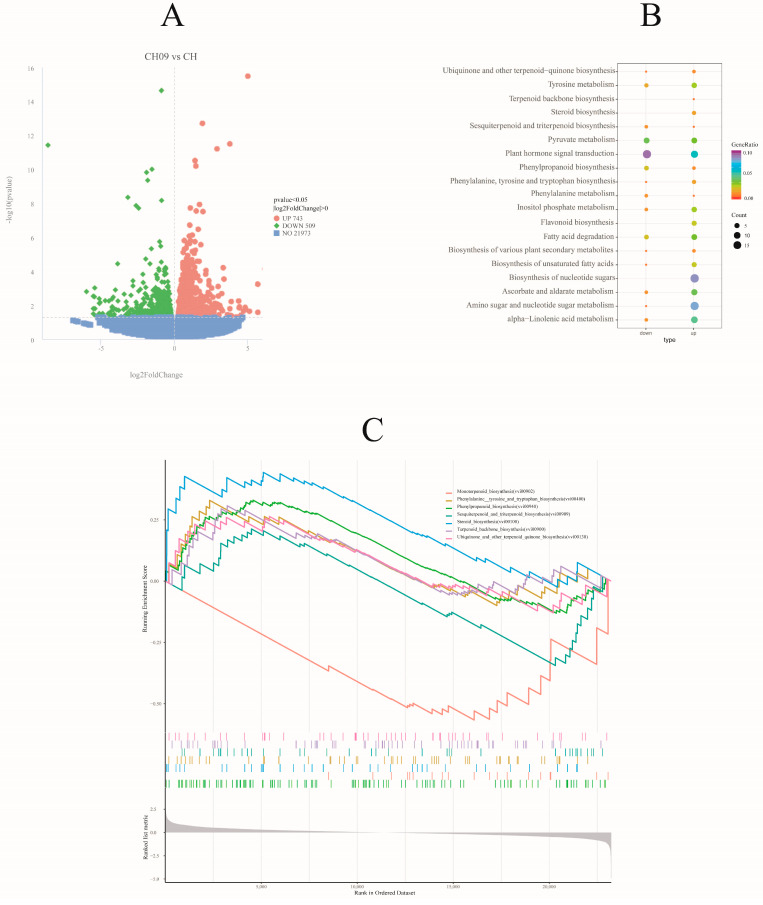
The volcano plot of up−(red) and downregulated (green) DEGs in CH09 vs. CH grape fruits (**A**), the KEGG pathway enrichment bubble chart (**B**), and the GSEA analysis results of KEGG pathway (**C**).

The standards of |log2FC ≥ 0| and adjusted *p* < 0.05 were used as criteria for the identification of the DEGs. We identified a total of 1251 DEGs from the comparison of CH09 vs. CH, with 743 genes upregulated and 509 genes downregulated (Figure 4A). Thus, there was a significant difference at the transcriptional level between CH and CH09 fruits at the mature stage.

#### 2.3.2. GO and KEGG Function Annotations of DEGs

Next, GO and KEGG pathway analysis tools were applied to the identified DEGs to gain insight into gene functionality. The significantly enriched GO terms in molecular function were “xyloglucan: xyloglucosyl transferase activity,” “transferase activity,” transferring hexosyl groups,” and “glucosyltransferase activity” (Table S6). To further identify the biological pathways of activity in grape berries, KEGG enrichment analysis was carried out. The DEGs were enriched in 102 signaling pathways. Among them, there was an enrichment of six genes in pathways related to terpene synthesis, including sesquiterpenoid and triterpenoid, steroid, and terpenoid backbone biosynthesis pathways (Figure 4B, Table 1). Phenylalanine metabolism; phenylalanine, tyrosine, and tryptophan biosynthesis; and phenylpropanoid biosynthesis were also enriched (Figure 4B), with 11 DEGs in these pathways (Table 1). 

**Figure 5:**
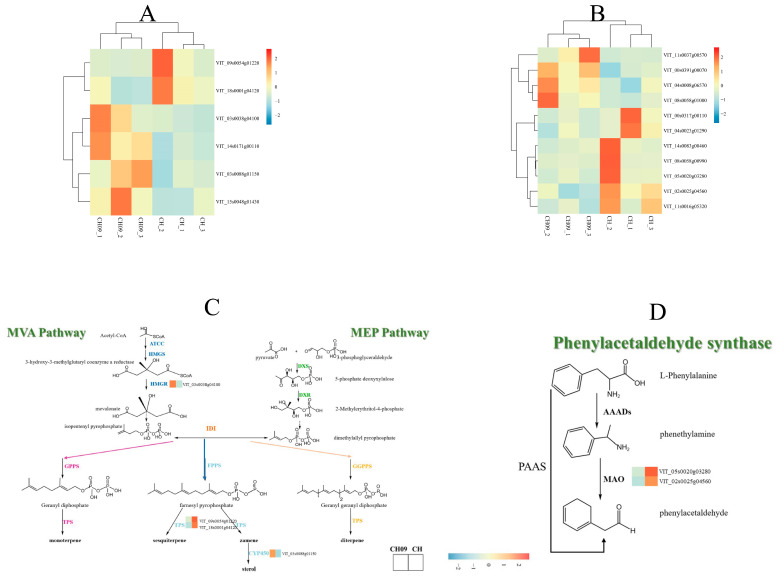
The terpene synthesis-related gene heat map (**A**), phenylalanine metabolism-related gene heat map (**B**), the schematic diagram of terpene synthesis pathway and the expression heat map of terpenoid synthesis pathway genes in CH09 and CH (**C**), and the schematic diagram of phenylacetaldehyde synthesis pathway and the expression heat map of phenylacetaldehyde synthesis pathway genes in CH09 and CH (**D**).

Different gene expression levels were observed in these metabolic pathways for CH09 and CH (Figure 5A,B). Compared with CH, VIT_03s0038g04100 (*HMGR*), VIT_14s0171g00110 (*LIP2*), VIT_03s0088g01150 (*CYP450*), VIT_15s0048g01430, VIT_11s0037g00570 (*SHT*), VIT_00s0391g00070 (*SHKB*), VIT_04s0008g06570 (*CM2*), and VIT_08s0058g01000 (*ASP1*) exhibited high expression in CH09. Among these highly expressed genes, only *HMGR* acts upstream of terpene synthesis, so changes in the expression of this gene may affect the synthesis of monoterpenes. In addition, terpenoid synthase (*TPS*) is the key enzyme in the final step of terpenoid synthesis. In the KEGG enrichment of DEGs, two *TPSs* were identified, VIT_09s0054g01220 (*TPS1*) and VIT_18s0001g04120 (*TPS2*). These genes are lowly expressed in CH09 and are mainly involved in the synthesis of sesquiterpenoid and triterpene biosynthesis. We also found that VIT_05s0020g03280 (*MAO1*) and VIT_02s0025g04560 (*MAO2*) were significantly downregulated at the transcriptional level (*p* < 0.05).

To further investigate the importance of metabolic pathways related to terpene and phenylalanine, the gene expression patterns of these pathways were obtained by heat map analysis (Figure 5C,D).

#### 2.3.3. Gene Set Enrichment Analysis (GSEA)

The traditional enrichment analysis of differentially expressed genes, such as KEGG and GO analysis, does not reveal the comprehensive regulation of pathways. Determining the upstream inputs and downstream effects is essential to fully understand the complexity of pathway regulation, as small changes in the levels or activities of upstream genes may lead to significant changes in the levels or activities of downstream genes. Additionally, if a fixed threshold is used for screening, some genes may not be identified, limiting the accuracy of enrichment analysis [16,17]. To gain more insight than that provided by traditional enrichment methods, we next performed gene set enrichment analysis (GSEA) to look at the expression of different genes in a pathway and the overall expression trend of the pathway [18]. 

The GSEA results based on the KEGG pathway of the mutant showed the significant downregulation of the monoterpene biosynthesis pathway and phenylalanine-related genes (Figure 4C). These results suggest that the monoterpenes of CH09 are not synthesized by the monoterpene biosynthesis pathway but may be regulated by other terpenoid biosynthesis-related genes. This analysis also supports the finding that *HMGR* is a key gene for monoterpene formation in CH09.

### 2.4. Correlation Analysis between Related Genes in the Terpene Metabolic Pathway and Terpene Substances

**Figure 6:**
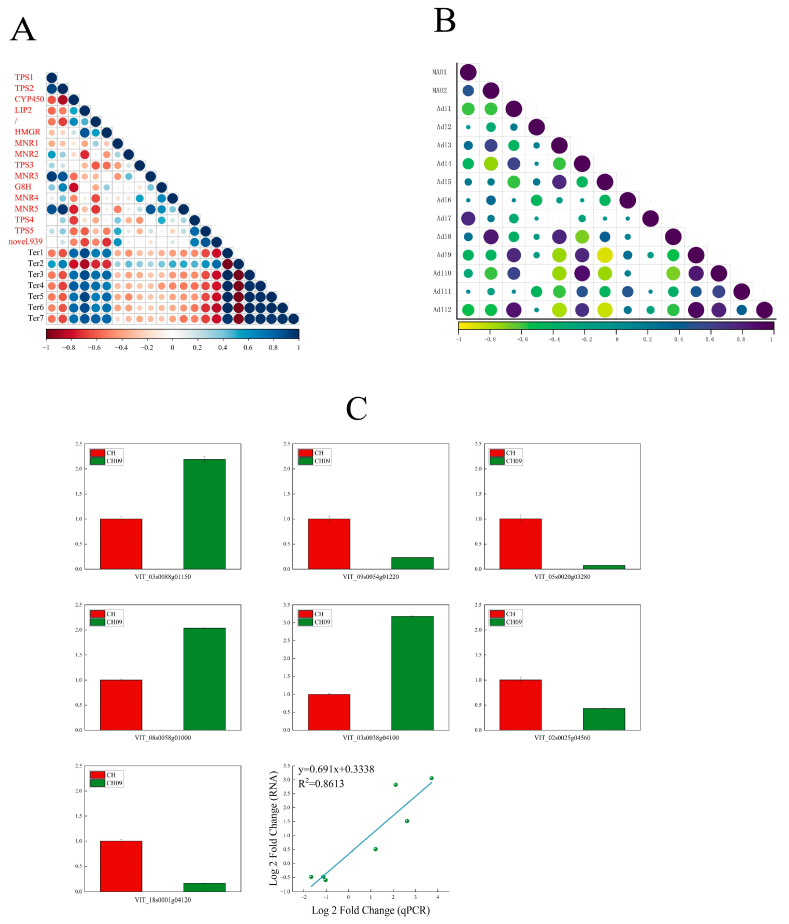
The correlation analysis of terpenoids and terpenoid synthesis−related genes in grapes (**A**); the correlation analysis of genes related to aldehyde and aldehyde compound synthesis in grape fruit (**B**); and the qRT−PCR validation of seven genes related to VOC biosynthesis in CH and CH09 and the correlation analysis of qRT−PCR and RNA−seq (**C**).

To explore the relationship between genes in metabolic pathways and terpenoids based on the key genes obtained from GO, KEGG, and GSEA results, the correlation between genes enriched in terpenoid biosynthesis and terpenoid volatiles was analyzed for CH09 and CH (Figure 6A). Linalool and geraniol, as the main fragrances of CH09, were significantly negatively correlated with genes *TPS1*, *TPS2*, *TPS5*, and *novel.939*, but significantly positively correlated with *HMGR* and *CYP450*. This indicates that these genes play a key role in the synthesis of linalool and geraniol. We analyzed the correlation between *MAO1*, *MAO2*, and aldehydes and found that these two genes were negatively correlated with phenylacetaldehyde (Figure 6B).

A set of DEGs identified in this research was selected for qRT-PCR verification. The qRT-PCR and transcriptome data showed a high correlation (Figure 6C), supporting the reliability of our RNA-seq analysis results. 

## 3. Discussion

### 3.1. Differences in Characteristics of Bud Mutation Variety

Compared with conventional hybridization breeding, bud sport is a consequence of genetic variation in somatic cells leading to the occurrence of qualitative and quantitative phenotypic alteration in plants, which can be observed in many vegetatively propagating plants, including grapes [19]. We studied the biological traits, basic physical and chemical indexes, and nutritional indexes of Chardonnay and bud sport. Considering the above point of view, the results were different from those of previous studies [19,20,21]; that is, it was difficult to find the difference between CH09 and CH only from the external phenotype.

The volatile compounds of CH and CH09 were qualitatively and quantitatively studied by SPME-GC-MS, and OPLS-DA and PCA results showed significant differences in the types and concentrations of VOCs between CH and CH09. Terpenes, especially monoterpenes such as geraniol and linalool, were almost exclusively clustered in CH09 (Figure 3D), and these compounds are the main contributors to muscat aroma [22,23]. In addition, phenylacetaldehyde, which has a desirable rose aroma [24], was detected in CH09 but not in CH. Consistent with previous findings, the analysis showed that Chardonnay is mainly composed of aldehydes and alcohols, compounds that typically lack unique aroma [25]. These results indicated linalool, geraniol, and phenylacetaldehyde were identified as the main VOCs and important biomarkers. Compared with Chardonnay, the aroma characteristics of CH09 were similar to those of muscat varieties, consistent with this bud mutant having a muscat aroma. There were no significant differences in other indicators, indicating that the mutant largely retained the characteristics of the parent Chardonnay. A similar analysis of ‘Kyoho’ and its early ripening bud mutant ‘Fengzao’ showed differences in the maturity stage and berry diameter but no significant differences in other biological traits [26]. Similarly, ‘Jinzao Wuhe’ maintained the early maturity and seedless traits of the parent ‘Himord Seedless’ but had larger fruits than the parent [20]. The phenomenon of grape bud mutation is quite common, with the maintenance of the original genetic structure. These results indicate that bud mutations of different varieties are diverse.

### 3.2. The Key Genes of Muscat Aroma in Bud Mutant

In order to clarify the molecular mechanism of aroma differences between Chardonnay and bud mutants, the main metabolic pathways and potential key genes of aroma differences between CH and CH09 fruits were explored by transcriptome sequencing. GO analysis revealed enrichment in glycosyltransferase and other pathways. Monoterpene compounds in grapes are present in free and bound glycoside forms, which are interconvertible [27]. The glycosylation reaction is mediated by glycosyltransferases (GTs), which catalyze the transfer of activated nucleotide sugars (such as UDP glucose) to the receptor glycoside ligand to form O-, S- and N-glycosides [28]. GTs can catalyze the glycosylation modification reaction and change the solubility and storage of terpene metabolites [29]. VvGT14 can catalyze the glycosylation of linalool, geraniol, and nerol to form the corresponding glycoside compounds [30]. Glycoside-bound monoterpenes can also be hydrolyzed into free compounds in developing berries, thus becoming a potentially important source of berry aroma [31]. Thus, the enrichment of GTs suggests a mechanistic basis for the formation and loss of grape rose aroma. To clarify the molecular mechanism of aroma differences between Chardonnay and the bud mutant, the main metabolic pathways and the potential key genes of aroma differences between CH and CH09 fruits were explored by transcriptome sequencing. *HMGR* is a key enzyme in the MVA pathway, which is mainly involved in the synthesis of sesquiterpenes and triterpenes. *HMGR* can have different effects on the aroma formation of grape varieties with different aroma types [32]. *VvHMGR3* improved the types of aroma components in strawberry-flavor grape, with significant increases in linalool, α-terpineol, and β-pinene content [33]. *VvHMGR3* is linked with the genes in the carotenoid biosynthesis and MEP pathways, which can interact with MVA kinases [34]. The expression of *HMGR* was positively correlated with the aroma intensity of muscat grape [23]. In this study, the KEGG enrichment and expression pattern analysis showed that *HMGR* was upregulated in CH09. However, no other sesquiterpenes and triterpenes were found in CH09, and instead, monoterpenes were prominent. This is similar to the above findings that there is an exchange between the MVA and MEP pathways. MVA is the main pathway for the synthesis of CH09 monoterpenes, with *HMGR* playing an important role. In addition, we found that two monoamine oxidase genes *MAO1* and *MAO2* were downregulated in CH09, which would seem inconsistent with the high content of phenylacetaldehyde in CH09. Phenylalanine can be directly catalyzed by phenylacetaldehyde synthases (PAASs) to form phenylacetaldehyde in the main pathway of phenylacetaldehyde biosynthesis. Phenylalanine can also form phenylethylamine under the catalysis of aromatic amino acid decarboxylases (AAADs), and phenylethylamine can form phenylacetaldehyde under the catalysis of MAO, but the formation of phenylacetaldehyde through this pathway is very limited [35]. The low expression of *MAO1* and *MAO2* could change the biosynthesis pathway of phenylacetaldehyde, with the direct conversion of phenylalanine into more phenylacetaldehyde. Similarly, Libin Sun et al. (2021) found that a low expression of *MAO* inhibited the formation of phenylacetaldehyde from phenylethylamine but promoted the degradation of phenylalanine to directly form phenylacetaldehyde, resulting in an increase in the phenylacetaldehyde content in Lentinula edodes [13]. GO and KEGG were screened by fixed thresholds, so some genes may not have been identified, limiting the accuracy of enrichment analysis. Therefore, GSEA and correlation analysis were carried out. The overall downregulation of the monoterpene biosynthesis pathway and the positive correlation between linalool, geraniol, and *HMGR* showed that *HMGR* acts as a key gene regulating the synthesis of monoterpenes in CH09. Studies have shown that the transient expression of *HMGR* in tomato increased the content of nerol by 5.7 times and linalool by 1.8 times [36]. The genes directly involved in monoterpene synthesis were found to be downregulated in the GSEA; however, we measured high levels of monoterpenes in CH09. These results indicate that there is an exchange between the MVA and MEP pathways, such that MVA is the main pathway for the synthesis of a large number of monoterpenes in the bud mutant, and *HMGR* is the key to the synthesis of monoterpenes in this pathway. Further analysis will be required to carefully determine the function of the identified genes and pathways and clarify their direct effects on monoterpene content.

## 4. Materials and Methods

### 4.1. Materials 

The experiments were performed at the Saishang Jiangnan winery in Ningxia (38°06′ N, 105°56′ E). Three-year-old CH and CH09 plants were used as experimental materials. The bud sport emerged from one branch of the Chardonnay plants, and berries were enriched with muscat flavor. The plant spacing was 3.0 m × 1.0 m, in the north–south direction, with a hedge frame and a ‘factory’ shape. Vines were managed with standard cultivation techniques, such as pruning, yield requirements, and harvest standards.

After the fruit was ripe (90 days after flowering), 10 clusters of each sample were randomly selected to measure the cluster weight and longitudinal and transverse diameters. Samples of similar size and lacking evidence of damage from pests or diseases were selected from the upper, middle, and lower parts of the cluster. In total, 6 to 8 berries were collected from each cluster, and 30 berries were randomly selected for the determination of single berry weight, as well as the longitudinal and transverse diameters. After measurement, fruit samples were frozen in liquid nitrogen and stored at −80 °C for subsequent analysis.

Additional details about the materials used for bud mutation identification are listed in Table S7.

### 4.2. DNA Extraction and ISSR Amplification

DNA extraction was performed using a Tiangen DNA secure plant genomic DNA extraction kit. The quality and quantity of DNA were detected by 1% agarose gel electrophoresis and NanoDrop, respectively. The dilution of each DNA sample (until reaching a concentration of 20 ng/μL) was performed according to the concentration of each DNA sample. Extracted genomic DNA of genotypes was amplified by PCR (polymerase chain reaction) using nine ISSR primers, which are listed in Table S8. The parameters of the PCR reaction system are described in Table S9, and amplification in a thermal cycler was performed using the settings described in Table S10. The products obtained from the PCR were detected by 1.0% agarose gel electrophoresis. 

### 4.3. Characteristic Evaluation 

The determination of single berry weight, longitudinal diameter, and transverse diameter was performed according to Wang [37]. The determination of total soluble solids (TSSs), titratable acid (TA), reducing sugar, total phenolic, and tannin was performed as previously described [38]. 

### 4.4. Extraction of Volatile Metabolites

The determination of volatile compounds was performed as described in [5], with slight modification. The grape was ground into 50 g homogenate in liquid nitrogen (during which 5 g PVPP was added). After soaking for 2.5 h, the supernatant was separated through centrifugation at 8000 rpm for 10 min. Next, 2 mL of grape juice was added to a headspace bottle containing 1.5 g of NaCl, and 40 μL (394 g/L) of 4-methyl-1-pentanol was added before the bottles were sealed. All samples were uniformly vibrated with a magnetically heated agitator at 40 °C set to 30 min. 

HS-SPME was performed using automatic injection by AOC-6000 (Shimadzu, Kyoto, Kyoto). The sample was activated for 3 min before injection, and the activation temperature was 280 °C. After activation, the extraction head was automatically inserted into the sample headspace bottle and extracted at 40 °C for 20 min. Desorption for 3 min was used for GC-MS separation and identification.

GC–MS analysis was performed by separating the desorbed volatiles in an Agilent TQ8050 (Agilent, Santa Clara, CA, USA) gas chromatograph with the following conditions: chromatographic column, DB-WAX-U1 (30 m × 0.25 mm, 0.25 μm) (Agilent, Santa Clara, CA, USA); carrier gas, He; flow rate, 37 mL/min, split injection, with a split ratio chromatographic column flow rate of 1 mL/min; and a purge flow rate of 6 mL/min. The column oven temperature program was 68 min in length, with an initial temperature of 40 °C, for 1 min, followed by an increase to 130 °C at a rate of 3 °C/min, and then an increase to 250 °C at a rate of 4 °C/min for 8 min. The ion source was EI, and the ion source and interface temperature were 200 °C and 250 °C, respectively. The solvent delay time was 1.5 min, the start time was 2 min, and the end time was 67.5 min. The acquisition mode was Q3 full scan, with start at 35 *m*/*z* and end at 500 *m*/*z*.

Qualitative and quantitative analyses of volatile metabolites were performed. VOCs were identified using the NIST 14 standard spectral library and further verified with retention indices (RIs) of Alkanes C8 to C40 (Sigma-Aldrich, Shanghai, China) on the DB-Wax column (30 m × 250 μm × 0.25 μm). The RIs were compared with those of the literature using the same column, and the same compound was identified as having an RI difference of five or less.

The peak area of each metabolite was normalized using the percentage of the identified component peak area in the area of the internal standard component multiplied by the inner standard mass.

The odor activity value (OAV) is widely used to evaluate the contribution of aroma components and is the ratio of the actual concentration of an aroma compound to the odor threshold [39]. In this study, OAVs were calculated for individual components by dividing the concentration by the previously reported odor threshold [11,27,40].

### 4.5. RNA Extraction and Sequencing Analysis

RNA extraction and the sequencing analysis of CH and CH09 were performed by Novogene Co., Ltd., Beijing, China (https://magic.novogene.com/, accessed on 1 September 2023). Sequencing libraries were generated using NEBNext^®^ Ultra™ II RNA Library Prep Kit for Illumina^®^ (NEB #E7775L). PCR was performed with random oligonucleotides as primers and the polymerase provided in the NEB kit. HISAT2v2.0.5 was used to construct the index of the reference genome. FeatureCounts (1.5.0-p3) was used to calculate the FPKM of each gene by normalizing the read count based on gene length and the total number of mapped reads. The key candidate genes were selected for qRT-PCR verification. The gene-specific primers used in the analysis were synthesized by Sangon Biotech (Shanghai, China), and the sequences are listed in Supplementary Table S11. Actin was used as a reference gene to normalize all gene expression levels by the previous reports. The 2^−ΔΔCt^ method was used to calculate the relative expression levels of genes.

### 4.6. Data Statistics and Analysis

All experimental data in this work are expressed as mean ±standard deviation (SD) for three biological replicates. The *t*-test was used to calculate the differences as *p*-values using SPSS26.0 (IBM Corporation, Endicott, NY, USA). Orthogonal partial least squares-discriminant analysis (OPLS-DA) was performed using SIMCA 14.1 software, and predictions were calculated. SPSS 26.0 software was used for single-factor analysis, and *p* < 0.05 and VIP ≥ 1 were used to screen the different aroma components.

## 5. Conclusions

Compared with Chardonnay, the kinds and total content of volatile compounds were significantly lower in the bud mutant, but there were significantly higher numbers and concentrations of terpenes and aldehydes, especially linalool, geraniol, and phenylacetaldehyde. Transcription sequencing and correlation analysis revealed *HMGR*, *TPS1*, *TPS2*, *TPS5*, *novel.939*, and *CYP450* as the key genes involved in terpene synthesis. The downregulation of *MAO1* and *MAO2* reduced the conversion rate of phenylethylamine to phenylacetaldehyde but favored the main synthesis pathway of phenylacetaldehyde, that is, phenylalanine degradation directly to phenylacetaldehyde, thus explaining the high content of phenylacetaldehyde in the mutant. These findings provide experimental data and valuable insights into cultivating clonal varieties of Chardonnay to maintain the quality and quantity of aromatic compounds for wine production and can guide molecular marker development. The next step will be to functionally verify the screened key candidate genes and develop functional markers to determine the molecular mechanism of muscat aroma synthesis.

## Figures and Tables

**Table 1:** Key candidate genes.

Gene ID	KEGG Description	Protein Names	Enzyme Type
*VIT_09s0054g01220*	Sesquiterpenoid and triterpenoid biosynthesis	Terpene cyclase/mutase family member	TPS1
*VIT_18s0001g04120*	Sesquiterpenoid and triterpenoid biosynthesis	Valencene synthase	TPS2
*VIT_03s0038g04100*	Terpenoid backbone biosynthesis	3-hydroxy-3-methylglutaryl coenzyme A reductase	HMGR
*VIT_14s0171g00110*	Steroid biosynthesis	Lipase	LIP2
*VIT_03s0088g01150*	Steroid biosynthesis	Squalene monooxygenase	CYP450
*VIT_15s0048g01430*	Steroid biosynthesis	Sterol methyltransferase C-terminal domain-containing protein	/
*VIT_15s0046g03590*	Monoterpenoid biosynthesis	(+)-neomenthol dehydrogenase	MNR1
*VIT_15s0046g03580*	Monoterpenoid biosynthesis	(+)-neomenthol dehydrogenase	MNR2
*VIT_06s0009g01450*	Monoterpenoid biosynthesis	(+)-neomenthol dehydrogenase	MNR3
*VIT_15s0046g03570*	Monoterpenoid biosynthesis	(+)-neomenthol dehydrogenase	MNR4
*VIT_06s0061g00670*	Monoterpenoid biosynthesis	(+)-neomenthol dehydrogenase	MNR5
*novel.939*	Monoterpenoid biosynthesis	(+)-neomenthol dehydrogenase	MNR6
*VIT_00s0372g00020*	Monoterpenoid biosynthesis	Terpene synthase metal-binding domain-containing protein	TPS3
*VIT_13s0067g00050*	Monoterpenoid biosynthesis	Terpene synthase N-terminal domain-containing protein	TPS4
*VIT_13s0067g00090*	Monoterpenoid biosynthesis	Terpene synthase metal-binding domain-containing protein	TPS5
*VIT_02s0012g02340*	Monoterpenoid biosynthesis	Geraniol 8-hydroxylase	G8H
*VIT_11s0037g00570*	Phenylpropanoid biosynthesis	Spermidine hydroxycinnamoyl transferase	SHT
*VIT_00s0391g00070*	Phenylalanine, tyrosine, and tryptophan biosynthesis	Phospho-2-dehydro-3-deoxyheptonate aldolase	SHKB
*VIT_04s0008g06570*	Phenylalanine, tyrosine, and tryptophan biosynthesis	Chorismate mutase	CM2
*VIT_08s0058g01000*	Phenylalanine, tyrosine, and tryptophan biosynthesis	Aspartate aminotransferase	ASP1
*VIT_00s0317g00110*	Phenylpropanoid biosynthesis	Serine aminopeptidase S33 domain-containing protein	CSE
*VIT_04s0023g01290*	Phenylpropanoid biosynthesis	Glycosyltransferase	GT5
*VIT_14s0083g00460*	Phenylalanine, tyrosine, and tryptophan biosynthesis	tryptophan synthase	trpB2
*VIT_08s0058g00990*	Phenylpropanoid biosynthesis	Peroxidase	PNC1
*VIT_05s0020g03280*	Phenylalanine metabolism	Amine oxidase	AOC1
*VIT_02s0025g04560*	Phenylalanine metabolism	Amine oxidase	AOC2
*VIT_11s0016g05320*	Phenylpropanoid biosynthesis	Peroxidase	PER25

## Data Availability

The data presented in this study are openly available in SRA at https://submit.ncbi.nlm.nih.gov/subs/, accessed on 1 June 2024, reference number SRP507154.

## Supplementary Materials

The following supporting information can be downloaded at: https://www.mdpi.com/article/10.3390/molecules29153671/s1, Figure S1: Amplification map of different primers in five test materials and Dendrogram of UPGMA cluster analysis. Figure S2: Leaf comparison between CH and CH09. Table S1: CH and CH09 fruit economic traits. Table S2: List of ISSR primers used in the study, number of band classes generated, and the fingerprint of each sample. Table S3: The composition and contents of the identified VOCs in CH09 and CH. Table S4: OAV of terpenes and phenylacetaldehyde. Table S5: Transcription sequencing data quality control analysis. Table S6: GO pathway analysis. Table S7: Information of accessions studied in this research. Table S8: List of ISSR primers used in the study, number of band classes generated, and the fingerprint of each sample. Table S9: PCR reaction system. Table S10: PCR amplification procedure. Table S11: The primers for qRT-PCR.
