# Supplementary material for: Transcriptome and Metabolome Analyses of Aroma Differences between Chardonnay and a Chardonnay Bud Sport
Source: Molecules. 2024 Aug 2;29(15):3671. doi: 10.3390/molecules29153671 (PMC11314234; doi:10.3390/molecules29153671)
Supplement: Supplementary file 1 [file molecules-29-03671-s001.zip › molecules-3057345-supplementary.pdf]

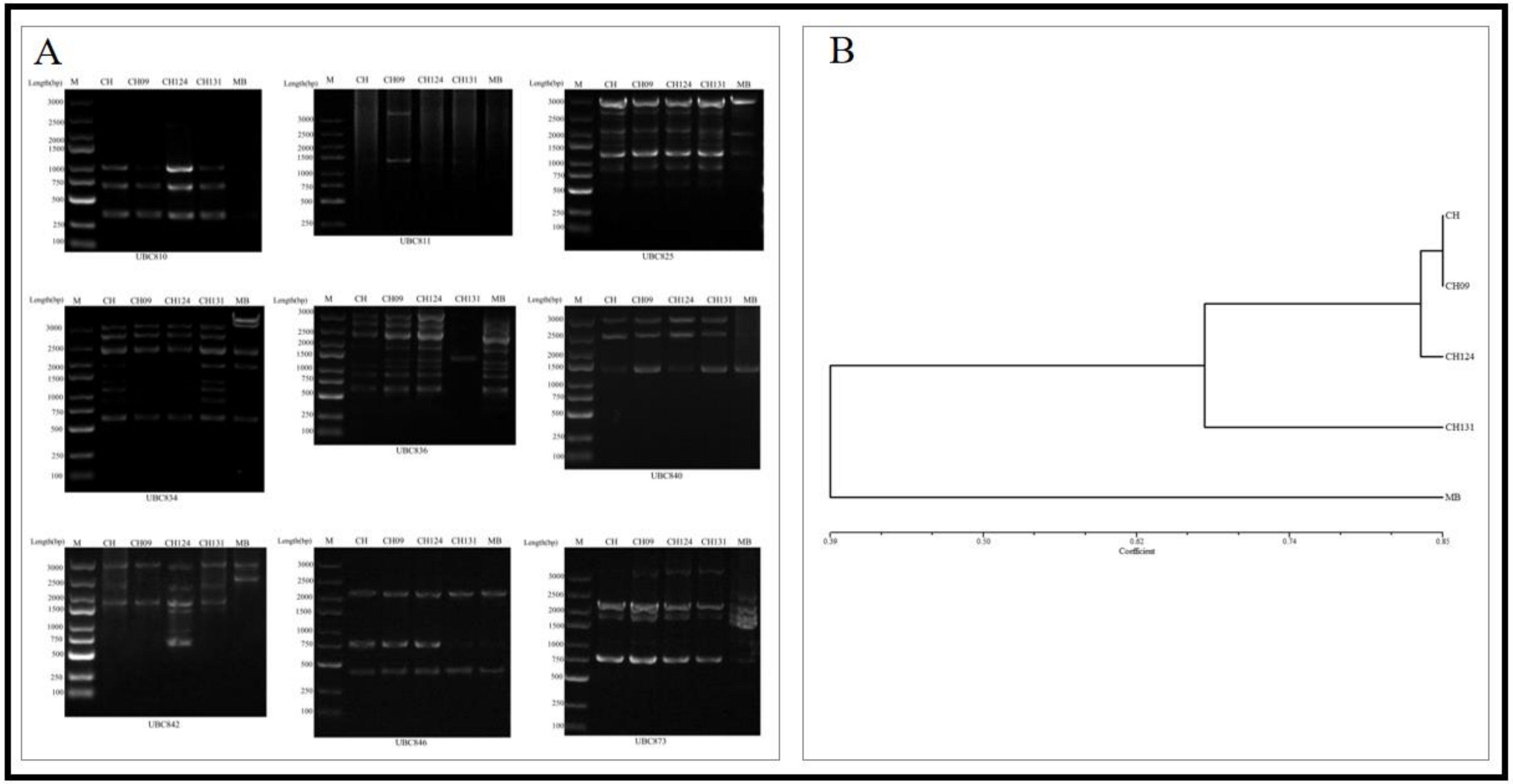

Figure S1. Amplification map of different primers in 5 test materials (**A**), Dendrogram of UPGMA cluster analysis (**B**). M represents DNA marker, CH represents Chardonnay, CH09 represents bud sport of Chardonnay, CH124 represents Chardonnay Nutrition 124, CH131 represents Chardonnay Nutrition 131 and MB represents Muscat Blanc.

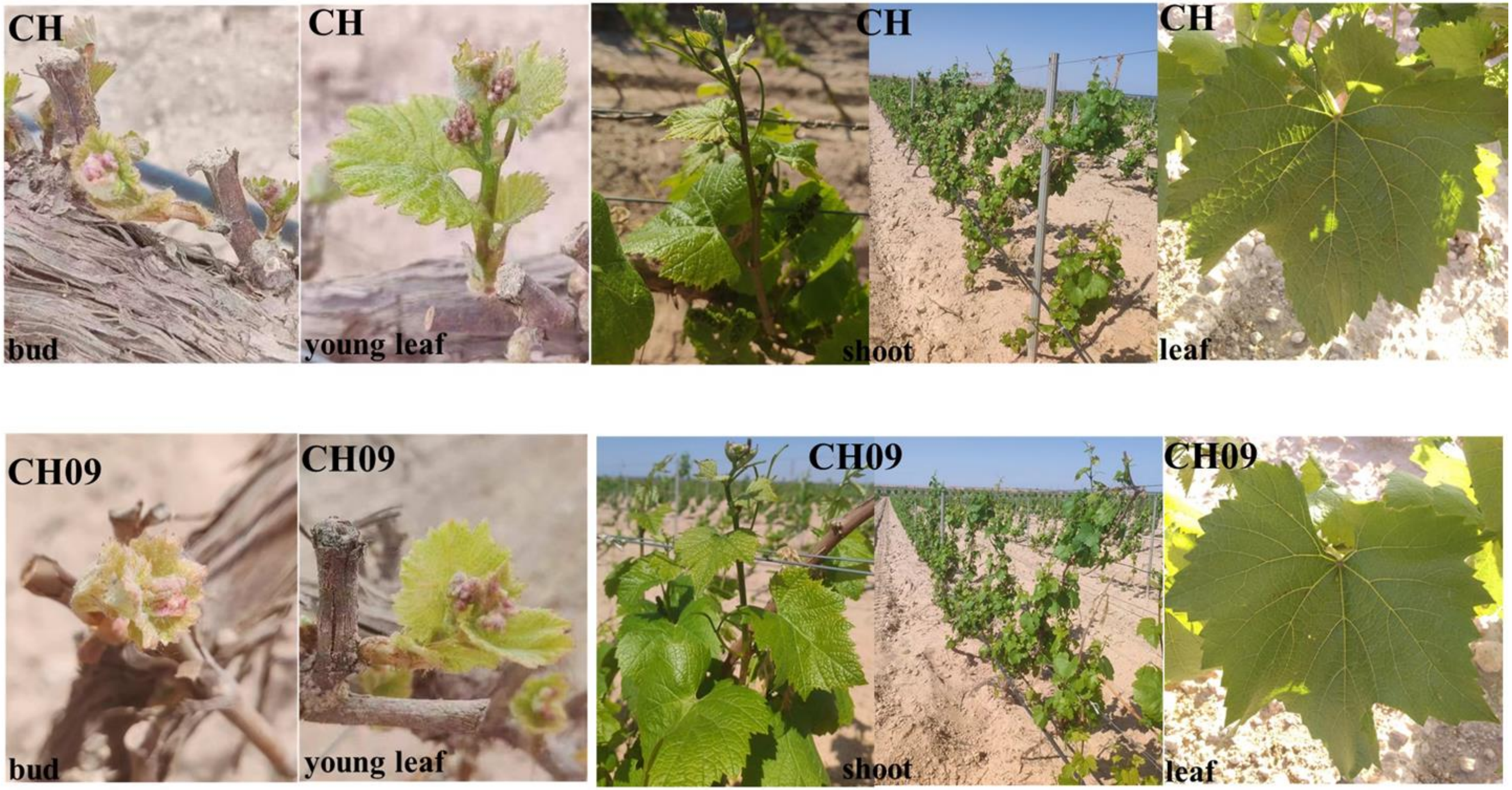

Figure S2. Leaf comparison between CH and CH09. CH represents Chardonnay, CH09 represents bud sport of Chardonnay

Table S1 CH and CH09 fruit economic traits

Variety	CW (g)	CLD (mm)	CTD (mm)	SW (g)	BLD (mm)	BTD (mm)
CH09	112.40±15.96a	124.10±4.22b	75.30±3.73a	1.70±0.20a	10.97±0.69a	10.96±0.64a
CH	128.96±18.26a	135.90±3.30a	77.90±2.22a	1.53±0.26b	10.54±0.98a	10.51±0.98b

Note: CW represents cluster weight, CLD represents cluster longitudinal diameter, CTD represents cluster transverse diameter, SW represents single weight, BLD represents berry longitudinal diameter, BTD represents berry transverse diameters. Different small letters means significant difference at $p < 0.05$. The same below.

Table S2 Fruit quality of CH and CH09

Variety	TSS (°Brix)	Total acid (g/L)	Reducing sugar (g/L)	Peel total phenol (mg/g)	Seed total phenol (mg/g)	Peel tannin (mg/g)	Seed tannin (mg/g)
CH09	19.33±0.52a	7.31±0.38a	184.30±2.08b	59.81±2.38a	104.76±1.15a	22.38±0.65a	34.94±0.12a
CH	20.0±0.63a	6.88±0.29a	192.17±2.02a	58.17±1.17a	103.49±0.511a	20.01±0.33b	34.12±0.65a

Table S3 The composition and contents of the identified VOCs in CH09 and CH.

RT (min)	MF[a]	Name [b]	Abbreviation [c]	Contents (μg/L) [d]		VIP [e]
				CH09	CH	
11.22	98.2	N-eicosane	Alk1	1.99±0.13	nd	1.041
13.3	97.56	3,5-dimethyloctane	Alk2	nd	28.00±1.99	1.040
15.87	96.63	2,5-Dimethylundecane	Alk3	2.80±0.06	6.31±0.19	1.042
16.98	92.70	3-Ethyl-3-methylheptane	Alk4	10.53±0.31	nd	1.044
18.45	98.58	2,6-Dimethylnonane	Alk5	nd	20.38±1.48	1.040
18.74	89.41	2,5-Dimethylnonane	Alk6	nd	5.05±0.18	1.044
18.96	92.44	3,6-Dimethyldecane	Alk7	nd	15.53±0.55	1.044
13.95	98.94	3,3-Dimethyloctane	Alk8	14.37±0.54	29.69±1.98	1.027
27.12	89.57	n-Hexadecane	Alk9	nd	26.10±1.48	1.042
11.34	90.22	4-Methyldodecane	Alk10	nd	6.31±0.36	1.042
19.14	90.71	5-(2-methylpropyl) nonane	Alk11	nd	62.47±5.10	1.039
20.42	88.45	Tetradecane	Alk12	nd	7.03±0.52	1.040
25.44	96.22	n-Pentadecane	Alk13	2.27±0.15	nd	1.042
31.13	90.09	n-Heptadecane	Alk14	12.01±0.39	9.82±0.87	
10.62	87.78	2-Methyl-1-penten-3-one	Ket1	nd	1.74±0.16	1.036
11.86	90.41	3-Hydroxy-2-butanone	Ket2	1.54±0.04	nd	1.045
14.87	95.45	Methylheptenone	Ket3	3.30±0.23	7.47±0.43	1.032
17.41	98.22	Sec-Octanone	Ket4	nd	1.31±0.05	1.044
18.73	99.12	2,5,5-trimethyl-2-hexene	Ter1	1.16±0.04	nd	1.044
19.88	98.30	Farnesene	Ter2	nd	8.41±0.38	1.043
19.52	97.79	Laurene	Ter3	3.21±0.20	nd	1.041
21.76	94.10	Nerolidol	Ter4	5.35±0.34	nd	1.041
42.55	87.55	Citronellol	Ter5	1.81±0.13	nd	1.041
44.89	93.23	Geraniol	Ter6	22.98±0.19	15.08±0.56	1.040
35.36	85.12	Linalool	Ter7	18.08±1.41	8.73±0.57	1.019
29.62	93.02	(*E, E*) -2,4-Hexadienal	Adl1	26.61±1.00	18.89±0.86	1.016
30.11	90.22	trans-2-nonenal	Adl2	0.86±0.03	0.85±0.05	
32.4	96.72	trans-2-decenal	Adl3	nd	4.27±0.09	1.045
18.65	99.09	3-Hexenal	Adl4	27.26±1.56	16.73±0.27	1.023
34.79	87.33	Benzaldehyde	Adl5	5.15±0.15	6.52±0.35	
25.01	94.51	n-Octylaldehyde	Adl6	1.60±0.03	1.61±0.07	
29.51	87.00	Nonanal	Adl7	3.66±0.27	3.70±0.18	
26.61	97.57	(E) -2-Heptenal	Adl8	15.01±0.21	103.65±2.24	1.045
38.88	89.94	Phenylacetaldehyde	Adl9	21.80±1.17	nd	1.042
26.23	90.33	Heptanal	Adl10	7.30±0.47	nd	1.041
33.77	90.45	Decanal	Adl11	1.13±0.02	1.06±0.07	
12.65	95.65	Isovaleraldehyde	Adl12	60.46±3.42	nd	1.042
14.89	92.34	2-Butyl-1-octanol	Alc1	nd	3.84±0.25	1.041

Table S3 (contuine)

RT (min)	MF[a]	Name [b]	Abbreviation [c]	Contents (μg/L) [d]		VIP [e]
				CH09	CH	
22.15	93.98	Trimethylsilanol	Alc2	nd	76.81±2.87	1.044
6.99	90.17	4-Methyl-2-pentanol	Alc3	nd	6.17±0.28	1.043
20.32	93.44	(+) -neomenthol	Alc4	nd	12.37±0.63	1.043
21.13	96.4	3,7-Dimethyl-6-octen-1-ol	Alc5	nd	1.84±0.10	1.043
35.12	87.89	cis-α, α-5-trimethyl-5-vinyltetrahydrofuran-2-methanol	Alc6	nd	1.41±0.06	1.044
15.77	89.21	Cyclooctanol	Alc7	nd	18.72±0.79	1.044
45.83	94.56	Benzyl alcohol	Alc8	6.49±0.51	3.86±0.22	1.002
13.1	97.45	Isoamyl alcohol	Alc9	6.67±0.27	nd	1.044
13.98	89.67	2-Ethylhexanol	Alc10	0.99±0.02	1.71±0.11	1.024
28.08	85.45	Trans-3-hexen-1-ol	Alc11	6.43±0.53	29.66±2.42	1.034
20.78	99.34	1-nonanol	Alc12	2.88±0.22	nd	1.039
11.38	86.47	Ethanol	Alc13	351.61±19.89	288.82±14.73	
19.22	94.55	1-penten-3-ol	Alc14	10.10±0.33	18.70±0.40	1.042
30.15	92.76	Terpineol	Alc15	5.79±0.33	nd	1.042
26.16	98.76	n-Heptanol	Alc16	2.87±0.11	3.85±0.33	
25.97	97.56	1-pentanol	Alc17	nd	15.31±0.98	1.041
31.64	93.98	1-Octen-3-ol	Alc18	11.67±0.48	75.44±5.26	1.038
30.17	90.69	Propylene glycol methyl ether	Oth1	3.40±0.17	19.35±1.14	1.040
28.34	89.45	2-pentylfuran	Oth2	3.05±0.04	nd	1.045

Note: [a] Match factor; [b] Volatile compound; [c] Abbreviations of VOCs in this study; [d] nd: Not determined; [e] Volatile compounds VIP ⩾ 1.

Table S4 OAV of terpenes and phenylacetaldehyde

CAS [a]	Name [b]	Abbreviation [c]	Threshold value [d]	OAV [e]
40467-04-7	2-Hexene	Ter1	uk	uc
502-61-4	Farnesene	Ter2	uk	uc
123-35-3	beta.-Myrcene	Ter3	36	ex
106-25-2	2,6-Octadien-1-ol	Ter4	300	ex
106-22-9	Citronellol	Ter5	10	ex
106-24-1	Geraniol	Ter6	7.5	3.06
78-70-6	Linalool	Ter7	0.5	36.16
122-78-1	Benzeneacetaldehyde	Adl9	4	5.45

Note: [a] CAS number; [b] Volatile compound; [c] Abbreviations of VOCs in this study; [d] uk: the threshold is unknown; [e] uc and ex: OAV cannot be calculated and OAV is less than 1, respectively

Table S5 Transcription sequencing data quality control analysis

Sample	Raw bases	Clean bases	Errorrate	Q20	Q30	GC content（%)	Multiple map (%)	Uniquely map (%)
CH_1	6.64	6.44	0.03	97.37	93.07	46.16	2.96	86.12
CH_2	6.65	6.06	0.03	97.73	93.83	46.55	3.27	87.01
CH_3	6.66	6.3	0.03	97.37	93.05	46.24	3.11	85.87
CH09_1	6.67	6.04	0.03	97.69	93.73	46.64	3.26	86.36
CH09_2	6.68	6.1	0.03	97.43	93.21	46.56	3.21	85.36
CH09_3	6.69	6.31	0.03	97.32	93	46.55	2.98	86.52

Table S6 GO pathway analysis

GO ID	Description	padj	Gene count
GO:0016762	xyloglucan: xyloglucosyl transferase activity	0.000	12
GO:0016758	transferase activity, transferring hexosyl groups	0.002	50
GO:0030246	carbohydrate binding	0.002	29
GO:0046527	glucosyltransferase activity	0.002	30
GO:0001871	pattern binding	0.002	17
GO:0030247	polysaccharide binding	0.002	17
GO:0016614	oxidoreductase activity, acting on CH-OH group of donors	0.012	26
GO:0004024	alcohol dehydrogenase activity, zinc-dependent	0.029	5
GO:0051903	S-(hydroxymethyl)glutathione dehydrogenase activity	0.029	5
GO:0016616	oxidoreductase activity, acting on the CH-OH group of donors, NAD or NADP as acceptor	0.029	23
GO:0016857	racemase and epimerase activity, acting on carbohydrates and derivatives	0.029	7
GO:0004022	alcohol dehydrogenase (NAD) activity	0.034	5
GO:0042562	hormone binding	0.038	8
GO:0004553	hydrolase activity, hydrolyzing O-glycosyl compounds	0.050	33

Table S7 Information of accessions studied in this research

Cultivar or strain	Abbreviations	fruit characters	Parents	characteristic aroma
Chardonnay	CH	The cluster is small and compact ; the berry is small, round and golden yellow.	Pinot Noir×Gouais	Green, grass, fruit
Muscat-like aroma mutant	CH09	The cluster is small and compact ; the berry is small, round and golden yellow.	Chardonnay	Rose, floral, geranium
Chardonnay Nutrition 124	CH124	The cluster is medium and loose ; fruit grains are conical, greenish yellow	Chardonnay	Almond, citrus fruit
Chardonnay Nutrition 131	CH131	The cluster is medium and loose ; fruit grain conical, yellow	Chardonnay	Melon, fig, fruit salad
Muscat Blanc	MB	The cluster is medium and very tight ; fruit grain medium, nearly round, greenish yellow	Unknown	Rose

Table S8 List of ISSR primers used in the study, number of band classes generated and the fingerprint of each sample

**Primer**	Squences (5'-3')	Number of amplified loci	Number of polymorphic loci	Polymorphic site ratio	CH	CH09	CH124	CH131	MB
UBC810	GAGAGAGAGAGAGAGAT	4	4	100%	0111	0111	1111	0111	0000
UBC811	GAGAGAGAGAGAGAGAC	2	2	100%	00	11	00	00	00
UBC825	ACACACACACACACACT	8	5	62.50%	11111111	11111111	11111111	11111111	10100100
UBC834	AGAGAGAGAGAGAGAGYT	9	6	66.67%	011111101	011110001	011110001	011111111	110011001
UBC836	AGAGAGAGAGAGAGAGYA	10	10	100%	11011101111	11011101111	11011101111	0000010000	0111101111
UBC840	GAGAGAGAGAGAGAGAYT	3	2	66.67%	111	111	111	111	001
UBC842	GAGAGAGAGAGAGAGAYG	7	6	85.71%	1011000	1001000	1011111	1011100	1100000
UBC846	CACACACACACACACART	3	1	33.33%	111	111	111	111	101
UBC873	GACAGACAGACAGACA	7	7	100%	0011001	1011001	1011001	1011001	0111110
Total		53	43	81.13%					

Note: Y=C or T；R=A or G

Table S9 PCR reaction system

Reagent name and concentration	Add the sample volume（μL）
2×Rapid Taq Master Mix	10
primer	2
DNA	1
ddH_2O	7
Total	20

Table S10 PCR amplification procedure

Temperature (°C)	Time	Cycle number (times)
95℃	3 min	1
95℃	15 s	
55℃	15 s	35
72℃	2 min	
72℃	10 min	
4℃		∞

Table S11 The primers for qRT-PCR

Gene ID	Forward primer	Reverse primer
VIT_03s0088g01150	AATGGAACAAGGAACGGTGAC	CTGGAGTGGGATAGGGAGAGG
VIT_09s0054g01220	ATGTGGTGCTACTGTCGGAT	ATGTGTTTCATTGTTACTTC
VIT_05s0020g03280	CTCCAGCCACAACGTAACCT	TGGGTTTGACTGTGGAGTCG
VIT_18s0001g04120	CCCCAGTTGCTAATCCAGA	AGACTCCTTGAACCTTCCCT
VIT_08s0058g01000	TGCTGAGTTTCAGAGGCGTT	CCCTGGAACTTGGGCATCTC
VIT_03s0038g04100	CCAACTCATAATCCACACAC	GAAAAATACACGAACCCATA
VIT_02s0025g04560	ACATGGATGGTGTGTTCGCT	TTCGGTCTCACCTCTCGGAT
Actin	TACAATTCCATCATGAAGTGTGATG	TAGAAGCACTTCCTGTGAACAATG
